# The Effects of Self-Perceived Parenting Attitudes on Visuo-Spatial Attention and Mental Rotation Abilities among Adolescents

**DOI:** 10.3390/ijerph19148841

**Published:** 2022-07-21

**Authors:** Sangyub Kim, Yeonji Baik, Kichun Nam

**Affiliations:** 1 Wisdom Science Center, Korea University, Seoul 02841, Korea; sangyub0310@gmail.com; 2 College of Interdisciplinary Studies in Cultural Intelligence, Dongduk Women’s University, Seoul 02748, Korea; ybaik@dongduk.ac.kr; 3 School of Psychology, Korea University, Seoul 02841, Korea

**Keywords:** adolescent, parenting attitude, visuo-spatial attention ability, mental rotation ability

## Abstract

**Highlights:**

- Self-perceived parenting attitude of adolecents affects their cognitive performances
- Adolescents’ self-perceived negative parenting attitudes lead to reduced visuo-spatial attention ability
- Adolescents’ self-perceived negative parenting attitudes induce employment of an impatient strategy for mental imagery

**Abstract:**

The present study aimed to investigate the effect of adolescents’ perceived negative evaluation of parenting on their visuo-spatial attention and mental rotation abilities. The useful field of view (UFOV) and mental rotation tasks were used to measure visuo-spatial attention and mental rotation abilities among adolescents. The experimental groups were divided into the negatively evaluating group (*M_Age_* = 18.44, SD = 0.87, 20.7% girls) and positively evaluating group (*M_Age_* = 18.40, SD = 0.81, 23.3% girls) based on their scores on the self-perceived parenting attitude scales. The UFOV task showed lesser accuracy of the negatively evaluating group when compared to the positively evaluating one in target perception presented in 20° visual angle, indicating a deteriorated visuo-spatial attention ability in the negatively evaluating group. In the mental rotation task, the negatively evaluating group exhibited a small trade-off effect between response times and rotation angles, which implied an impatient strategy was employed to perform the task, whereas such a trade-off was not observed in the positively evaluating group. Thus, both experimental groups differed in terms of their visual attention and mental spatial abilities. This study suggests that the reduced visuo-spatial attention and mental rotation abilities may act as precursors for serious psychological symptoms caused by the negative self-evaluation of their parents’ parenting attitudes.

## 1. Introduction

Research on adolescence has found that teenage years are characterized by the emergence of social and developmental problems, such as academic and school-adjustment problems, and conflicts with family and/or friends [1,2,3,4]. In particular, conflict with parents is one of the major problems that adolescents have to resolve, which will, in turn, allow them to develop social skills and prevent them from developing maladaptive problems [5,6,7]. There have been abundant research findings that suggest the positive effects of a positive parent–adolescent relationship on parent–adolescent communication and adolescents’ academic achievements [8], development of self-esteem [9,10], and mental health [11], which conclusively indicate close connections between parental relationship and rising problems during adolescence. For this reason, adolescents’ evaluation of their parents’ parenting attitudes, in turn, influences their emotional development, which may give rise to psychological symptoms, such as depression or aggression [12,13,14]. Authoritative parenting attitudes from the parents enable adolescents to form stable and positive relationships with their parents, which allows them to develop psychological and emotional stability [15,16,17]. On the other hand, parents’ authoritarian parenting attitudes can cause adolescents to cut off their relationship with their parents, which in turn leads to additional psychological and emotional problems [17,18,19,20]. Previous adolescent studies have also reported a positive association between emotional problems and cognitive impairments [21,22]. Matthews et al. [21] observed that depressed adolescent girls showed poorer performance relating to healthy controls in visual memory and spatial working memory tasks, indicating a positive relationship between depressive episodes and deteriorated cognitive functions related to visual and spatial working memory. Villemarette-Pittman et al. [22] employed a battery of verbal tests requiring a broad range of language and executive functions and revealed that individuals who exhibited impulsive aggression showed impaired executive functioning compared to nonaggressive controls. In addition, Hankin et al. [23] conducted a study involving pure depressed, pure anxious, and comorbid adolescents (ages 9–17 years) and reported that these adolescents showed attentional biases to sad and angry faces, whereas the control adolescents showed attentional avoidance of sad faces. Hence, previous literature suggests that emotional instability is closely related to cognitive decline among adolescents, and such emotional instability may be influenced by adolescents’ developing internal representations of their parents’ parenting attitudes, arising from negative parent–adolescent familial relationships.

In general, “parenting attitudes” refers to the attitudes adopted by parents who share their physical and psychological environments with their children on a daily basis, which, in turn, forms the core component of family relationships [24]. Expressing affection and enforcing discipline for children is important, as they learn socially appropriate behavior within the boundaries of their familial interactions [25]. Parents are often the first people to establish relationships with their children after they are born. Thus, they influence the formation of their children’s personalities and values. Moreover, previous studies of personality found evidence that descriptors of personality are able to change during adolescence, which suggests the flexible change of personality depending on experience with parents [26,27]. The personality and values of a child will affect the formation of their self-esteem in the future, and the self-esteem so formed determines whether they experience psychological symptoms, such as depression or anxiety [28,29]. Adolescents are still developing in terms of their cognitive abilities and are incomplete beings both socially and financially, as they are not yet independent from their parents. Thus, adolescence is a period in which the support of a caregiver, parents in most cases, is necessary to establish himself or herself as a member of society. Adolescents’ assessment on their parents’ attitudes may have more diverse effects on them (i.e., the quality of life, self-assessment, and social adjustment) even though parent–adolescent relationships are bidirectional, possibly because adolescents turn to their parents and internalize their parenting attitudes to form internal social figures of themselves. It indicates that the self-perceived parenting attitude of the adolescents influences their life regardless of their parents’ perception on parenting or even the actual abundant physical and spiritual contribution of their parents.

Therefore, the adolescents’ evaluation of parenting attitude can be considered as one of the central factors affecting emotional instability and a determining factor in the psychological symptoms experienced by adolescents.

In addition, these psychological symptoms may bring about changes in their cognitive abilities. Some studies have discussed negative effects of anxiety on the task performance requiring working memory [30], and others have reported cases of impaired spatial working memory among healthy individuals after they were exposed to an anxiety-provoking situation [31,32]. These results suggest that psychological symptoms are closely associated with cognitive declines, indicating potential impairment in cognitive abilities due to emotional instability instigated by psychological symptoms.

The manifestation of psychological symptoms and related cognitive impairments are expected to appear in adolescence and research findings suggest that such psychological manifestations observed in later adulthood may have been accumulated since early childhood [33,34,35,36]. In particular, according to OECD, only 55% of Korean adolescents in South Korea showed satisfaction in their living situation, which is significantly lower than that of other OECD countries (84.8% on average) [37]. It suggests that Korean adolescents are prone to be vulnerable to their emotional problems, such as depression and anxiety, expecting a more dramatic change in psychological symptoms and related cognitive abilities. Moreover, cognitive abilities can change with minor changes in psychological symptoms, but these minor changes may not meet the criteria for adolescents to be diagnosed with mental disorders. Previous literature on mild cognitive impairment (MCI) and dementia have reported cases of cognitive ability changes which emerged before the clinical diagnosis of mental disorders related to cognitive impairment. Bäckman et al. [38] and Ewers et al. [39] observed that people who are diagnosed with MCI and dementia showed cognitive declines for several years before they were clinically diagnosed, indicating that cognitive impairment might be considered a preclinical symptom of MCI or dementia. Thus, an adolescent who perceives one’s parents’ parenting attitudes negatively may also show cognitive decline before the actual appearance of obvious psychological symptoms.

In fact, while most research has targeted younger children, there is a lack of research on cognitive decline of adolescents with psychological symptoms [40,41,42]. The cognitive abilities of younger children have been measured indirectly through playful interactions that use picture storybooks, object detection, and virtual role play, which makes it hard to differentiate and measure the scope of sub-cognitive abilities (e.g., memory and attention) [24]. Therefore, it is essential to examine changes in specific sub-cognitive abilities according to the psychological symptoms among adolescents through cognitive tasks. The current study focused on the visuo-spatial attention and mental rotation abilities. The visuo-spatial attention is the set of a mechanism by which selectively focused visual target was pointed to specific objects, locations, or certain instants in time [43]. Thus, irrelevant visual information is restrained while relevant visual information is captured in spatially constrained visual acuity. According to research, declined visuo-spatial attention is a typical alteration of cognitive ability in a stress-induced situation [44,45]. It affects the formation of spatial representation in visual imagery processing based on objects and requires the involvement of primary and secondary visual cortices [46]. Decrease in visuo-spatial attention ability may induce deterioration of mental rotation ability.

Therefore, this study aims to understand whether dysfunctions in visuo-spatial attention and mental rotation abilities are evident among adolescents who have a negative evaluation of their parents’ parenting attitudes and whether their cognitive performance may serve as a cue to possible mental health disorders. It is reasoned that adolescents who evaluate parenting attitudes negatively may exhibit reduced visuo-spatial attention and mental rotation abilities when compared to those who evaluate their parents’ parenting attitudes positively. Thus, the current study was planning to recruit adolescent participants and divide the participants into two groups, according to their evaluation of their parents’ parenting, and control their possible psychological symptoms (attention deficit hyperactivity, anxiety, depression, aggression, manic-depression) to examine the difference in visuo-spatial attention and mental rotation abilities.

## 2. Method

### 2.1. Participants

A total of 67 adolescents were recruited from Korean high schools by an advertisement of the participant recruitment, and they agreed to participate in this study. Among them, 8 participants were excluded from the final data analysis due to their failure to follow the direction of an experimenter and comply with the experimental procedures. The participants were categorized into the two experimental groups, according to their evaluation of their parents’ parenting attitude, with a median split method, as the present study targeted the effect of their evaluation of their parents’ parenting on visuo-spatial attention and mental rotation abilities. We used the scores on the father’s parenting attitude to assign the participants to each experimental group even though both of the father’s and mother’s parenting attitude were measured. You and Yi [47] reported that the perceived parenting attitudes of adolescences were more highly correlated for fathers than mothers. In addition, Kim [48] showed paternal rejection indifference and hostile attitude more significantly explained symptoms of depression and externalizing problems of adolescent male students, considering with the proportion of male participants in this study (about 78% of the total participants; 46 males, 13 females). Using the participants’ evaluation on their father’s parenting attitude is more appropriate to determine the criterion only for the experimental group classification as their father’s parenting attitude has a strong positive correlation with their mother’s parenting attitude (*r* = 0.629, *p* < 0.001). Eventually, the negatively perceiving group reported low scores of both the father’s and mother’s parenting attitudes; on the contrary, the positively perceiving group showed high scores of both the father’s and mother’s parenting attitudes. Thus, data from 29 (23 males, 6 females) negative and 30 (23 males, 7 females) positive evaluation groups were analyzed. The average ages of the negatively and positively perceiving groups were 18.44 (*SD:* 0.87, range: 17–19 years) and 18.40 years (*SD:* 0.81, range: 17–19 years), respectively. This study followed the ethical standards laid down in the 1964 Declaration of Helsinki. All participants comprehended the ethics code and provided written consent with their parents’ permission toward their engagement in this study. A small amount of money was paid as a reward for their participation. Participants were excluded if they: (1) had a known history of neurological impairment because of a stroke or brain damage, (2) had been diagnosed with an intellectual disability, (3) had a known history of impairment of major sensory organs, (4) had a known history of substance abuse or gambling addiction, (5) were unable to participate voluntarily, (6) had severe medical conditions that had the potential to interfere with their ability to participate in the study.

To examine the negative effects of the psychological symptoms on their performance in the UFOV and mental rotation tasks, the participants were given self-reported psychological questionnaires after performing the UFOV and mental rotation tasks in order to measure the possibility of symptoms associated with attention deficit hyperactivity disorder (ADHD), depression, anxiety, aggression, and manic depression. Research has shown that the emotional state of an individual interacts with their cognitive abilities. For instance, decreased visuo-spatial attention ability has been associated with ADHD symptoms [49], anxiety and aggression [50,51,52], depression [53,54], and manic depression [55]. People with ADHD [56], anxiety [57], depression [58,59], aggression [60], and manic depression [61] showed declined mental rotation abilities.

Hence, self-reported psychological questionnaires (ADHD, BAI, BDI, K-AQ, and K-MDQ) were administered to the participants to measure the symptoms of the above-mentioned mental disorders. Finally, participants were given parents’ attitude evaluation questionnaires (PAT-F and PAT-M, Oh and Lee, [62]) to compare negative and positive evaluation groups. The negative evaluation group scored 187.76 (*SD:* 10.99) and the positive evaluation group scored 236.03 (*SD:* 16.81) on the father’s parenting attitude scale, which indicated a significant difference (*t*(57) = −13.005, *p* < 0.001). On the mother’s parenting attitude scale, the negative evaluation group scored 198.28 (*SD:* 23.43), whereas the positive evaluation group scored 228.67 (*SD:* 35.84), indicating a significant difference (*t*(57) = −3.841, *p* < 0.001). There were no significant differences in other self-reported psychological questionnaire scores between the experimental groups (*t*(57) = 1.603, *p* = 0.114 for ADHD; *t*(57) = 1.496, *p* = 0.140 for BAI; *t*(57) = 1.174, *p* = 0.245 for BDI; *t*(57) = 1.739, *p* = 0.087 for K-AQ; *t*(57) = 0.749, *p* = 0.457 for K-MDQ). The scores were not so severe that they had to be clinically diagnosed with mental disorders either. Table 1 shows all raw scores from the questionnaires.

### 2.2. Self-Report Psychological Questionnaires

The father’s (PAT-F) and the mother’s (PAT-M) parenting attitude scales were used to evaluate parenting attitudes as perceived by the adolescents. Psychological questionnaires on ADHD, BAI, BDI, K-AQ, and K-MDQ were given to participants to minimize the individual differences between groups that may be affected by other possible symptoms of mental disorders.

#### 2.2.1. Parent’s Attitude Evaluation Questionnaire

This study used Lee’s [63] PAT-F and PAT-M scales, which are the recent versions of the adolescent’s evaluation of their parents’ parenting attitudes, originally developed by Oh and Lee [62]. Each questionnaire was divided into 4 categories comprising 15 questions each, namely affection, autonomy, achievement, and rationality. Thus, they comprised 60 questions in all. The participants were instructed to respond to these questionnaires by choosing an option on a 5-point Likert scale that ranged from 1 = strongly disagree to 5 = strongly agree. The total score ranged from 60 to 300 points, where higher scores denoted greater positive evaluation of the perceived parenting attitudes.

#### 2.2.2. ADHD Questionnaire

It was originally developed by Conners [64] to measure ADHD symptoms as a self-reported questionnaire. It comprises three different versions for parents, teachers, and adolescents, and each has a regular (L) and short (S) form [65]. The standardized Korean version of adolescent short-form questionnaire, with a 4-point Likert scale (0 = disagree, 1 = slightly agree, 2 = agree, 3 = strongly agree), was adopted. It comprised 27 questions from 4 subscales (behavior and cognitive problems, hyperactivity, and ADHD indicators) (Bahn et al., 2001). The level of ADHD was assessed based on the total scores, which ranged from 0 to 81 points. If the total score exceeds 42 points, the respondent may be diagnosed with ADHD [66].

#### 2.2.3. Beck Anxiety Inventory (BAI)

The Korean version of BAI was used to measure the level of anxiety among participants [67]. It was originally developed by Beck et al. [68] and was used to assess an individual’s level of anxiety in this study. All responses were self-reported on a 4-point Likert-scale (I did not feel anxiety at all = 0; I felt a little anxiety = 1; I felt a lot of anxiety = 2; I felt severe anxiety = 3 points). The level of anxiety was evaluated by computing the total scores of 21 items, which ranged from 0 to 63 points. Higher total scores indicated higher levels of anxiety [67].

#### 2.2.4. The Korean Version of Beck Depression Inventory II

The extent of depressive disorder was measured using the Korean version of Beck Depression Inventory II [69]. This inventory was developed by Beck et al. [70] and was used to assess the severity of depressive symptoms among adolescents over the age of 13 years throughout adulthood. Participants had four options to choose from (I did not feel depression at all = 0; I felt some depression = 1; I felt quite a lot of depression = 2; I felt severe depression = 3). The total score for 21 questions indicated the level of depression, which ranged from 0 to 63 points. The criteria for being depressive was classified into 4 different levels (0–9 points: not depressive; 10–15 points: slightly depressive; 16–23 points: severely depressive; 24–63 points: extremely depressive) [71].

#### 2.2.5. Korean—Aggression Questionnaire (K-AQ)

The Korean version of the Aggression Questionnaire (K-AQ) was used to measure the level of aggression among the participants [72]. This questionnaire was developed by Buss and Perry [73]. It comprised four measurement sub-scales (levels of physical and verbal aggression, anger, and hostility). The participants had 5 options to choose from (not at all = 1; slightly agree = 2; agree = 3; usually agree = 4; absolutely agree = 5 points), and the total score for 27 questions ranged from 27 to 135 points. A higher total score indicates higher levels of aggression [72].

#### 2.2.6. Korean—Mood Disorder Questionnaire (K-MDQ)

The Korean version of the Mood Disorder Questionnaire was used to measure bipolar disorder among the participants [74,75]. The questionnaire was developed by Hirschfeld et al. [76]. Participants had to pick between two options (yes = 1; no = 0). The total score for 13 questions ranged from 0 to 13 points. A score of 7 points implied being potentially diagnosed with bipolar disorder [75].

### 2.3. Experimental Tasks

**Figure 1:**
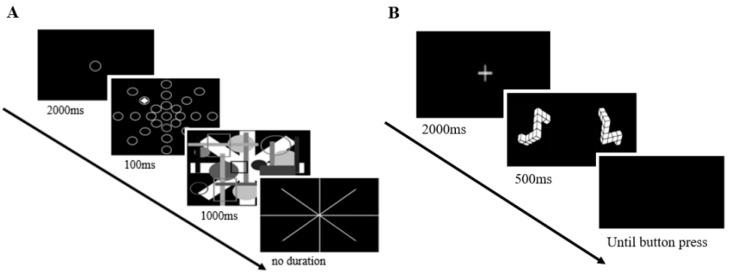
The experimental paradigms of (**A**) UFOV task and (**B**) mental rotation task are illustrated. (**A**) Participants had to identify the direction in which the target had been presented. (**B**) Participants had to determine whether the two given 3D-shaped targets were identical.

The first cognitive task used in this study was the useful field of view (UFOV) task, which has been typically used to measure visuo-spatial attention abilities [77,78]. Figure 1A presents the experimental procedure for this task. Instructions were given to the participants followed by a fixation point that lasted 2000 ms at the center of the computer monitor screen. After the fixation point was presented, target stimuli were presented for 100 ms in one of the 8 directional positions (“left-bottom,” “middle-bottom,” “right-bottom,” “left,” “right,” “left-top,” “middle-top,” and “right-top”). The experimental condition was the visual angle of the target in which it was presented. The target stimulus was presented at the viewing angles of 10°, 20°, or 30° from the center of the screen. Participants were instructed to respond by pushing the numeric pad of the keyboard as quickly and accurately as possible, according to the target positions presented regardless of the visual angle. For example, participants had to press number “3” on the numeric pad while responding correctly on the target stimulus presented at a 10° angle on the screen (“right-bottom” position). Similarly, number “7” had to be pressed while responding to the target stimulus presented at “left-top” at the 30° angle. A total of 3 practice trials were firstly given to the participants and then 120 main trials were presented, 40 stimuli in each experimental condition (3 visual angles).

The second cognitive task was the mental rotation task developed by Shepard and Metzler [79] to measure both mental rotation and visuo-spatial attention abilities [80,81,82]. Mental imagery refers to the ability to express the spatial and temporal state of an object accurately by forming the shape or movement of the object through visual representation [83]. It requires one to memorize the visual shape and spatial information of an object, and then transform its position by changing its mental image of the perceived object. In the experimental procedure of this task (Figure 1B), instructions were first given and a fixation point was presented for 2000 ms followed by the two 3D figures, which were displayed simultaneously on the left and right visual fields of the participants with computer monitor screen. They had to determine by pressing the keyboard whether the two 3D shaped targets were identically shaped objects as quickly and accurately as possible if the objects were rotated (slash (‘/’) button—two targets were identical, ‘z’ button—two targets were nonidentical). The experimental condition for this task comprised the rightward rotated-angles of the two presented figures (0°, 30°, 60°, 90°, 120°, 150°, and 180°), and 196 stimuli were created using 28 stimuli, 14 identical pairs, and 14 nonidentical pairs placed in 7 angles. Identical pairs were same in shape but nonidentical pairs were different. These different pairs were used to measure accurate responses with mental rotational representation related to spatial memory, which supplements the problem of response bias toward “Yes (two 3D targets are same in shape)” in identical pairs as Shepard and Metzler [79] only included identical target pairs for judgment, occurring response biases of judgments interfering with appropriate analyses on response times and accuracy rates.

### 2.4. Statistical Analysis

A separate two-way mixed ANOVA was performed on the accuracy rates and response times from UFOV and the mental rotation tasks. For UFOV, statistical analysis was conducted on the three visual angle conditions (10°, 20°, 30°) with groups (positively and negatively evaluating groups) as the between-subject variable. For the mental rotation task, statistical analysis was performed on seven rotation-angle conditions (0°, 30°, 60°, 90°, 120°, 150°, and 180°) with groups as the between-subject variable.

## 3. Results

This study investigated the changes in adolescents’ visuo-spatial attention and mental rotation abilities based on their evaluation of their parents’ parenting attitudes. The research question was whether the group that evaluated their parents’ parenting attitudes negatively would show a decrease in visuo-spatial attention and mental rotation abilities when compared with the one that evaluated parenting attitudes positively.

**Figure 2:**
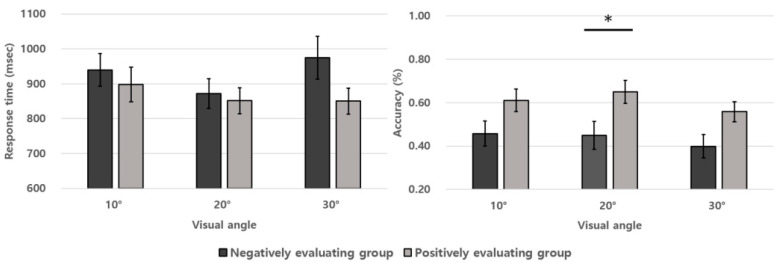
Response times and accuracy rates of two experimental groups in the performance of the UFOV task. Error bars represent the standard error. (* *p* < 0.05).

The results of the accuracy rates and response times in the UFOV task are described in Figure 2 and Table 1. A two-factor (group condition × visual angle condition) mixed ANOVA was carried out on the accuracy rates. There were significant main effects for the group and the visual angle conditions (*F*(1, 57) = 5.481, *p* = 0.023, $\eta_{p}^{2}$ = 0.088; *F*(2, 114) = 9.849, *p* < 0.001, $\eta_{p}^{2}$ = 0.147). The two-way interaction effect between group and visual angle conditions was not significant (*F*(2, 114) = 1.198, *p* = 0.306, $\eta_{p}^{2}$ = 0.021). The main effect of the two groups showed significantly lower accuracy rates in the negatively evaluating adolescent group than the positively evaluating one. However, The LSD post-hoc test on the main effect of the visual angle condition revealed significantly higher accuracy rates in 10° and 20° visual angles, as opposed to the 30° visual angle (*p* = 0.280 for comparison between 10° and 20°; *p* = 0.004 for comparison between 10° and 30°, *p* < 0.001 for comparison between 20° and 30°). Next, the two-factor mixed ANOVA was performed on the response times. It showed no significant main effects and the two-way interaction effect in the group and visual angle conditions (*F*(1, 57) = 1.312, *p* = 0.257, $\eta_{p}^{2}$ = 0.022 for the group main effect; *F*(2, 114) = 2.078, *p* = 0.130, $\eta_{p}^{2}$ = 0.035 for the visual angle main effect; *F*(2, 114) = 1.595, *p* = 0.207, $\eta_{p}^{2}$ = 0.027 for the two-way interaction effect of those two conditions).

**Figure 3:**
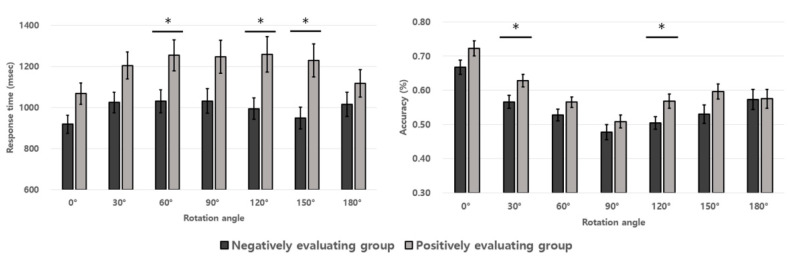
Response times and accuracy rates of two experimental groups in the performance of the mental rotation task. Error bars represent the standard error. (* *p* < 0.05).

The results of accuracy rates and response times in the mental rotation task are described in Figure 3 and Table 2. The two factor (group condition × rotation-angle condition) mixed ANOVA was performed on the accuracy rates of the identical pair judgments, not for nonidentical pairs. There were significant main effects of the group and the rotation angle conditions (*F*(1, 57) = 5.413, *p* = 0.024, $\eta_{p}^{2}$ = 0.087; *F*(6, 342) = 23.137, *p* < 0.001, $\eta_{p}^{2}$ = 0.289). The interaction effects between group and rotation angle conditions were not significant (*F*(6, 342) = 0.790, *p* = 0.578, $\eta_{p}^{2}$ = 0.014). As the results show, the main effect of group condition means lower accuracy rates in the negatively evaluating adolescent group than in the positively evaluating one. The LSD post-hoc test on the significant rotation angle condition revealed that higher accuracy rates were observed in smaller rotation-angle conditions. Next, the two factor (group condition × rotation-angle condition) mixed ANOVA was performed on the response times. There were significant main effects of the group and the rotation angle conditions (*F*(1, 57) = 6.016, *p* = 0.017, $\eta_{p}^{2}$ = 0.095; *F*(6, 342) = 6.416, *p* < 0.001, $\eta_{p}^{2}$ = 0.101). The two-way interaction effect between the group and the rotation angle conditions was significant (*F*(6, 342) = 2.278, *p* = 0.036, $\eta_{p}^{2}$ = 0.038). As the results show, the main effect of group condition indicates faster response times in the negatively evaluating adolescent group than in the positively evaluating one. In addition, the LSD post-hoc test on the significant main effect of the rotation angle condition showed faster response times in 0° than other rotation angle conditions (*p* < 0.001, *p* < 0.001, *p* < 0.001, *p* < 0.001, *p* = 0.001, *p* = 0.028). Moreover, faster response times in 90° than in 180° was shown (*p* = 0.031). Simple main effect analysis on the two-way interaction effect revealed significantly faster responses in the negatively evaluating adolescent group than the positively evaluating one in the response times in 0°, 30°, 60°, 90°, 120°, and 150° rotation angle conditions (*F*(1, 57) = 4.716, *p* = 0.034, $\eta_{p}^{2}$ = 0.076; *F*(1, 57) = 4.739, *p* = 0.034, $\eta_{p}^{2}$ = 0.077; *F*(1, 57) = 5.569, *p* = 0.022, $\eta_{p}^{2}$ = 0.089; *F*(1, 57) = 4.574, *p* = 0.037, $\eta_{p}^{2}$ = 0.074; *F*(1, 57) = 6.892, *p* = 0.011, $\eta_{p}^{2}$ = 0.108; *F*(1, 57) = 8.355, *p* = 0.005, $\eta_{p}^{2}$ = 0.023).

## 4. Discussion

This study examined the influences of adolescent evaluation of their parents’ parenting attitudes on visuo-spatial attention and mental rotation abilities. It examined changes in adolescents’ visuo-spatial attention and mental rotation abilities based on their evaluation of parenting attitudes while their self-reported psychological symptoms were controlled for ADHD, BAI, BDI, K-AQ, and K-MDQ. It sought to understand whether the group that evaluated parenting attitudes negatively would show declined visuo-spatial attention and mental rotation abilities when compared with the one that evaluated parenting attitudes positively.

The results of the UFOV task show that the negatively evaluating group had lower accuracy rates in 20° visual angle of the target, thus indicating deteriorated visuo-spatial attention ability. In the mental rotation task, the positively evaluating group showed a typical trade-off effect between response times and rotation angles (i.e., a linear relationship between the amount of rotation angles and increase in response time) as reported in Wilson et al. [84], whereas the negatively evaluating group performed in a less typical fashion by showing a small trade-off function. In addition, the negatively evaluating group had a tendency of lower accuracy rates and faster response times when compared to the positively evaluating one, indicating that more hasty responses were made in the former than in the latter. Generally, slower response times and higher accuracy rates were observed when the participants used conservative strategies while performing the tasks [85], suggesting that the negatively evaluating group may use an impatient strategy in the task performance due to deterioration of mental rotation ability.

On mental rotation task, negative evaluation group showed rather atypical trade-off between accuracy rates and response times, suggesting different strategic planning in forming internal representation of objects. According to Wilson et al. [84], children with developmental coordination disorder (DCD) encounter difficulties in producing accurate visuo-spatial representations, and a small trade-off function between response times and accuracy rates was observed among children with DCD when compared to the control group in the mental rotation task. Authors have concluded that children with DCD do not automatically use motor imagery but rather depend on the alternative object-based strategy to perform the task due to deficit of motor imagery. The small trade-off between accuracy rates response times among negative evaluation group may also rely on different cognitive strategies to perform the task due to their impairment in forming the internal representation of the object during mental rotation task.

Therefore, the results of this study suggest that the negative evaluation of parenting attitudes by adolescents may first induce a distinct decrease in cognitive abilities, such as visuo-spatial attention and mental rotation, before the actual diagnosis of related mental disorders takes place. It is a fact that cognitive abilities, such as emotions, do not change dichotomously but rather continuously from monotonous to dramatic, based on the psychological symptoms. This is the reason why cognitive deficits of visuo-spatial attention and mental rotation may precede the obvious diagnosis of psychological symptoms induced by the negative evaluation of parenting attitudes.

Why did we observe initial deterioration of both visuo-spatial attention and mental rotation abilities for adolescents who have rated negatively on their perception of their parents’ parenting attitudes? First, negative emotions, such as depression, anxiety, fear, and anger, induced by emotionally unstable states make it hard to control the appropriate information processing for a given set of stimuli [86,87,88,89]. When humans become emotionally unstable, the emotional state changes violently, and they react sensitively to the stimuli, which, in turn, leads to emotional instability [90]. This is called a “human defense mechanism” in psychology and helps avoid situations or stimuli while eliciting negative emotions within oneself [91]. It results in selective attention toward specific traits in the course of perceptual and cognitive information processing [92]. In this regard, Janelle et al. [44] investigated the change in attentional focus in the states of anxiety and excitement. They instructed their participants to detect cues related to the purpose of the experiment among several cues that were presented in the peripheral visual field during a virtual driving task. The participants encountered great difficulty in identifying the related cue from among the distractors, as their levels of anxiety and excitement rose. In addition, in their analysis of gaze behaviors, the participants with narrower attentional fields induced by high levels of anxiety were more disturbed by the irrelevant cues that were presented in the peripheral visual field. In addition, Pine et al. [93] found the significant attention avoidance of threatening faces of maltreated children relative to normal children by comparing the performances of a visual-probe task that manipulated the levels of threat. Their study also suggested the changes in attention to avoid negative emotion as a mechanism of defense.

Other research has also shown that negative emotions (i.e., anxiety) affect spatial working memory rather badly [31,32,94]. Lavric et al. [31] found poorer performance in the spatial working memory task in threat-provoking conditions relative to safe conditions when they assessed levels of anxiety through heart rate recordings and self-report scales. Li et al. [94] also reported similar effects of negative emotion on spatial working memory by using the event-related potential technique in a modified n-back task. The authors found greater P300 effect for negative emotion in the anterior and posterior areas, which reflect cognitive impairment in spatial working memory. Shackman et al. [32] found that threat-induced anxiety selectively disrupted the performance of spatial working memory but not verbal working memory. In addition, Taylor-Tavares et al. [95] observed an impairment in the spatial working memory and attentional shifting ability specifically in unipolar depression group relative to bipolar depression group. Hence, negative emotions induced by adolescent’s adverse perceptions of their parents’ parenting attitudes may potentially impair their ability to effectively use spatial working memory, which may have in turn led to a deterioration in their mental rotation ability.

Other studies have dealt with the relationship between experiences of stress in the early stage of life and the presence of subsequent psychiatric symptoms [96,97,98,99]. Experiencing stress early on in life may lead to the development of other psychiatric symptoms, such as anxiety, depression, post-traumatic stress disorder (PTSD), and substance abuse later in adulthood. Bremner and Vermetten [100] found changes in the neural underpinnings of related cognitive abilities after the revelation of psychiatric symptoms. In addition, there have been a series of papers that have reported the relationship between parenting stress and the existence of attention deficit hyperactivity disorder in their child [101,102], which may suggest a potential danger of stress to psychological states of both parents and their children. This supports the idea that stress that occurs among children as a result of inadequate parenting may produce cognitive deficits and psychological symptoms.

The deterioration of the visuo-spatial attention and mental rotation abilities may lead to alterations in other cognitive abilities, as cognitive domains of humans are interconnected [103,104,105]. Kim et al. [106] highlighted the possibility that declined visuo-spatial attention ability as a result of high-level social phobia induced emotional perception bias in the recognition of facial expressions. This suggests that changes in some cognitive abilities as a result of psychological symptoms can alter related ones. Thus, changes in cognitive abilities as a result of psychological symptoms can also be influenced by interactions among the cognitive abilities [60,106,107,108]. Emotional instability preceded by cognitive deficits (i.e., deteriorated visuo-spatial attention and mental rotation abilities) may impair other related cognitive abilities. This implies that decreased visuo-spatial attention and mental rotation abilities may be used as behavioral markers in understanding and diagnosing psychological symptoms.

The negative evaluation of parenting attitudes by adolescents resulted in the deterioration of visuo-spatial attention and mental rotation abilities among adolescents before they were diagnosed with associated mental disorders. These cognitive deficits may be an indicator of changes in psychological symptoms induced by the negative perception of parenting attitudes among adolescents, even before they are diagnosed with clinical mental disorders. Our results suggest that the cognitive decline observed among adolescents with a negative evaluation of parenting attitudes may act as a behavioral marker, and this may serve as prognostic factors influencing clinical outcome of psychological symptoms arising from emotional instability within a family. Future research should explore how the deterioration in visuo-spatial attention and mental rotation abilities, which are prognostic symptoms of possible psychological disorders, can affect other related cognitive functions, such as emotional perception bias and cognitive control.

Lastly, there are two limitations of this study. Firstly, the current study only revealed a few cognitive abilities. We only measure the visuo-spatial attention and mental rotation abilities of adolescents. However, the adolescents’ evaluation of their parents’ parenting is expected to have an effect on diverse cognitive abilities, such as spatial and verbal memory, attentional control, and executive function, which require further research regarding the effect of their evaluation on another cognitive ability. The second is the absence of parents’ own evaluation of their parenting. The parents’ own evaluation of their parenting is able to be used to examine the mediation of potential factors, which affect adolescents’ evaluation of the parenting. The mismatch between the parents’ own evaluation and adolescents’ evaluation of their parents’ parenting is critically affecting the emotional states or cognitive performances of adolescents since it is presumed to be a lack of communication between adolescents and their parents. We hope that future studies deal with these two issues to understand precisely the relationship between the adolescent and their parents’ parenting.

## 5. Conclusions

The current study investigated the effect of adolescents’ perceived negative evaluation of parenting on their visuo-spatial attention and mental rotation abilities. It showed that the negatively evaluating group showed less accuracy relative to the positively evaluating group in the UFOV task. Moreover, the negatively evaluating group exhibited a small trade-off effect between response times and rotation angles in the mental rotation task, implying the employment of the impatient strategy to perform the task. Therefore, this study found that the reduced visuo-spatial attention and mental rotation abilities in the negatively evaluating group in contrast with the positively evaluating group, suggesting the critical importance of parenting to adolescents in terms of cognitive development not only for emotional development. Furthermore, the findings of the current study implicate that the adolescent is able to perceive the parenting as bad even if their parents provide a good economical or material environment. It potentially leads to the deterioration of their cognitive ability due to a negative evaluation of their parents’ parenting. Therefore, it is important to provide a positive parenting environment through parent–adolescent communication other than the abundant physical and spiritual contribution of adolescents’ parents.

## Figures and Tables

**Table 1:** The accuracy rates and response times of the two experimental groups in the UFOV task. The value within brackets denotes the standard error.

	Visual Angle 10°		Visual Angle 20°		Visual Angle 30°	
	Acc	RT	Acc	RT	Acc	RT
Negatively evaluating group	0.46 (0.06)	940 (58)	0.45 (0.06)	872 (43)	0.40 (0.05)	974 (60)
Positively evaluating group	0.61 (0.05)	898 (35)	0.65 (0.05)	851 (36)	0.56 (0.05)	850 (35)

**Table 2:** The accuracy rates and response times for the two experimental groups in the mental rotation task. The values in parentheses denote the standard error.

	Degree of Rotation													
	0°		30°		60°		90°		120°		150°		180°	
	Acc	RTs	Acc	RTs	Acc	RTs	Acc	RTs	Acc	RTs	Acc	RTs	Acc	RTs
Negatively evaluating group	0.67 (0.02)	919 (43)	0.57 (0.02)	1025 (50)	0.53 (0.02)	1031 (55)	0.48 (0.02)	1032 (60)	0.50 (0.02)	994 (52)	0.53 (0.03)	949 (52)	0.57 (0.03)	1015 (59)
Positively evaluating group	0.72 (0.02)	1068 (52)	0.63 (0.02)	1204 (64)	0.57 (0.02)	1254 (76)	0.51 (0.02)	1246 (79)	0.57 (0.02)	1259 (85)	0.60 (0.02)	1228 (80)	0.58 (0.03)	1117 (64)

## Data Availability

The collected and analyzed data in this study are only available after permission from the authors if provided with an acceptable request.
